# Real-time ultrasound-guided thoracentesis in the intensive care unit: prevalence of mechanical complications

**DOI:** 10.1186/s13089-020-00172-9

**Published:** 2020-04-26

**Authors:** David Rene Rodriguez Lima, Andrés Felipe Yepes, Claudia Inés Birchenall Jiménez, Mario Andrés Mercado Díaz, Darío Isaías Pinilla Rojas

**Affiliations:** 1 Emergency Medicine and Critical and Intensive Care Medicine, Hospital Universitario Mayor Méderi–Universidad del Rosario, Bogotá, Colombia; 2 Critical and Intensive Care Medicine, Hospital Universitario Mayor Méderi–Universidad del Rosario, Bogotá, Colombia

**Keywords:** Ultrasound, Thoracentesis, Complications, Intensive care

## Abstract

**Background:**

The use of thoracic ultrasound during thoracentesis reduces complications. The aim of this study was to determine the prevalence of complications for real-time ultrasound-guided thoracentesis performed by intensivists. As a secondary objective, the change in oxygenation before and after the procedure was evaluated.

**Patients and methods:**

An observational prospective study was conducted. A total of 81 cases of real-time ultrasound-guided thoracentesis performed by intensivists in the intensive care unit (ICU) of Méderi Major University Hospital, Bogotá, Colombia, between August 2018 and August 2019 were analyzed. Thoracentesis performed by interventional radiologists and using techniques different from the focus of this study were excluded from the analysis.

**Results:**

There was one pneumothorax, for a prevalence rate of mechanical complications in this population of 1.2%. The mean partial oxygen pressure to inspired oxygen fraction ratio (PaO_2_/FiO_2_) prior to the procedure was 198.1 (95% CI 184.75–211.45), with a PaO_2_/FiO_2_ after the procedure of 224.6 (95% CI 213.08–226.12) (*p* < 0.05).

**Conclusions:**

Real-time ultrasound-guided thoracentesis performed by intensivists is a safe procedure and leads to a significant improvement in oxygenation rates. Future studies are required to determine the impact of these results on other outcomes, such as mortality, ICU stay, and days of mechanical ventilation.

## Background

Pleural effusion is a frequent finding in the intensive care unit (ICU), and it can cause hypoxemia and alterations in lung mechanics [1].

The prevalence of pleural effusion in the ICU can vary between 40 and 60% [2]. The commonly reported causes of pleural effusion in this population are infectious exudates (43%), non-infectious exudates (33%) and transudates (24%) [3]. In postoperative patients undergoing cardiovascular surgery, up to 7% present with pleural effusion, the most common cause being hemothorax in up to 50% of cases, with dyspnea as the predominant symptom [4].

Pleural effusions with documented volumes greater than 500 ml affect gas exchange, hemodynamic stability and respiratory work, and it has been demonstrated that drainage of pleural effusions in ICU patients under mechanical ventilation is related to improved oxygenation indices, increased end-expiratory volume and decreased transpulmonary pressure [5]. A recent meta-analysis that included 31 studies with 2265 patients showed that drainage of pleural fluid produces improvement in PaO_2_/FiO_2_ as an oxygenation index and tends to increase end-expiratory volume [6].

Thoracentesis is a percutaneous procedure for collecting pleural fluid, and it has diagnostic utility and therapeutic applications. It is recommended for pleural effusions of unknown cause, because it allows defining the cause of the effusion and has therapeutic utility in large-volume pleural effusions associated with respiratory distress [7]. Thoracentesis should not be performed for bilateral effusions in a clinical picture strongly suggestive of transudate (e.g., cardiac failure), unless the presentation is atypical or does not respond to clinical management [8, 9].

Complications related to the performance of blind thoracentesis include a high incidence of pneumothorax (11%) [10]; for this reason, the use of ultrasound guidance is strongly recommended for performing interventions in the pleural space and using small-diameter catheters [11, 12]. In turn, the diagnostic sensitivity of ultrasound for pleural effusion is higher compared to that of chest X-ray and allows identifying the pleural fluid characteristics that differentiate complicated and uncomplicated effusions and homogeneous and heterogeneous effusions [13]. In addition, the routine implementation of pulmonary ultrasound in the ICU decreases the number of chest X-rays, with a reduction in medical costs and radiation exposure, without affecting the clinical results [14].

Various techniques have been developed to estimate the volume of pleural fluid by ultrasound, with a good correlation between the drained liquid and that calculated prior to the procedure, finding that distances between the diaphragm and the visceral pleura greater than 30 mm are related to pleural effusions greater than 500 ml [15]. Balik et al. described a formula to calculate the pleural effusion volume by ultrasound by measuring the maximal interpleural distance (Sep) in millimeters (mm) in end-expiration at the lung base in a posterior axillary line and multiplying this value by 20, quantifying the pleural fluid volume (Vpl) in milliliters (ml) [16]. The method described by Balik et al. was validated in patients under invasive mechanical ventilation in a supine position with mild trunk elevation at 15°; however, the mean prediction error of this equation is high (158 ± 160 ml) [16, 17]. The position of the patient influences the volume calculated by this method because when elevating the headboard, the free pleural fluid experiences the effects of gravity and can increase the interpleural distance [18]. In Balik et al.’s original work, no difference in correlation of interpleural distance with the right- and left-side drained volumes was found [16]; however, other studies have found a better correlation on the right side [19, 20]. This is secondary to the fact that on the left side, the heart increases the level of a pleural effusion, like a stone in a container with water, leading to overestimation of the interpleural distance [18].

The aim of this observational study was to determine the prevalence of complications of real-time ultrasound-guided thoracentesis performed by intensivists. As a secondary objective, the change in oxygenation before and after the procedure was evaluated.

## Patients and methods

Méderi Major University Hospital (*Hospital Universitario Mayor Méderi*) is a Colombian high-complexity hospital with 780 hospital beds and 97 adult ICU beds where routine use of ultrasound for the performance of guided procedures by intensivists has been implemented for 5 years.

During a period of 13 months (between August 2018 and August 2019), data were collected prospectively from 81 consecutive real-time ultrasound-guided thoracentesis performed by intensivists in the ICU of Méderi Major University Hospital, Bogota, Colombia. Patients undergoing thoracentesis using methods other than real-time ultrasound-guided techniques and those in whom thoracentesis was performed by interventional radiologists were excluded from the analysis. Authorization for this study was granted by the Institutional Human Research Ethics Committee of Del Rosario University (DVO005 1068-CV1177).

All thoracentesis were performed by intensivists with more than 5 years of experience, who during their graduate education had at least 1 month of certified ultrasound training. The decision to perform thoracentesis was made only for clinical reasons and was not based on the protocol.

Pleural effusion is diagnosed by portable chest X-ray findings (anteroposterior view and supine position), pleural ultrasound or chest tomography according to the clinical applicability for each patient. If the treating physician considered the patient to be a candidate for drainage, an assessment was performed by an intensivist with experience in thoracic ultrasound. This professional performed the thoracic ultrasound, evaluating the chest wall in eight areas as recommended by the International Evidence-Based Recommendations for point-of-care lung ultrasound [21] to confirm the presence of pleural effusion and to determine whether it was susceptible to drainage.

Estimation of the pleural effusion volume (Vpl) in ml was performed similarly to the method described by Balik et al.: Vpl (ml) = Sep (mm) * 20; however, the majority of patients included in this study were not under mechanical ventilation and showed little tolerance to the supine position at 15°, and therefore, the investigators decided to perform the measurement with the trunk elevated to 30° regardless of whether the patient was under invasive mechanical ventilation or spontaneous breathing.

**Fig. 1:**
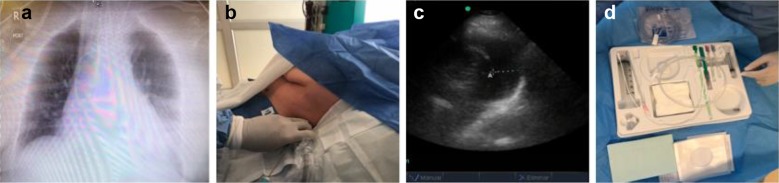
**a** Chest X-ray of the patient with left pleural effusion during the postoperative period of myocardial revascularization. **b** Patient in the supine position with the head at 30°; ultrasound assessment in the lower thorax on the posterior axillary line. **c** Quantification of the pleural effusion with a 5-MHz convex transducer; measurement of the distance between the visceral pleura and the posterior wall of the thorax in the transverse axis. In this case, the distance is 26 mm, with an approximate calculated volume of 520 ml by the Balik method. **d** Thoracentesis kit

**Fig. 2:**
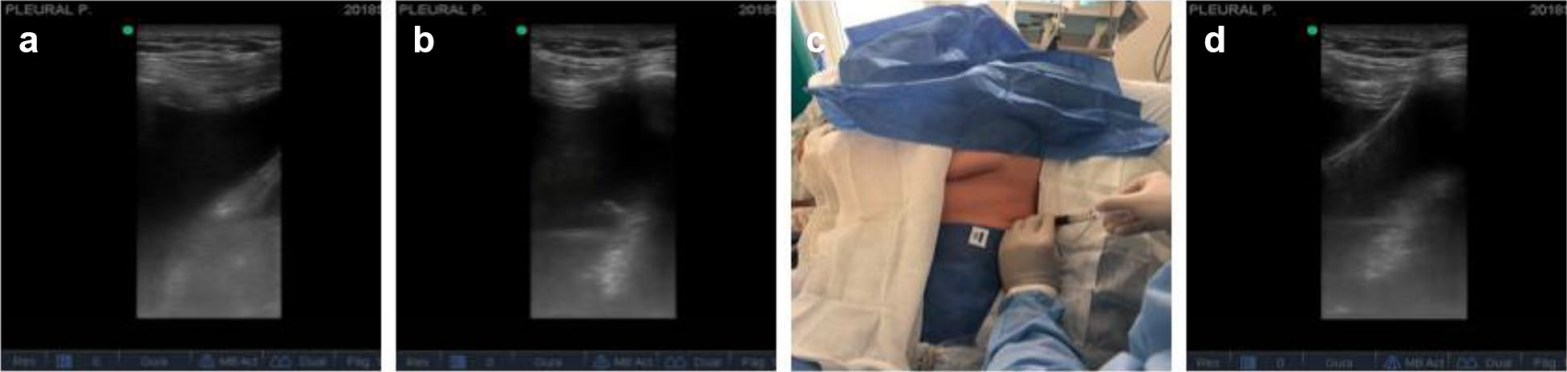
**a** Assessment, with a 13-MHz linear transducer, of the effusion in the lower aspect of the thorax on the posterior axillary line, visualizing the upper and lower ribs, intercostal space, pleural fluid, and diaphragm. **b** After verifying no interposition of vessels with color Doppler, needle puncture is performed; needle passage into the pleural cavity is visualized in real time. **c** Pleural fluid return is verified, in this case blood. **d** Passage of the guide toward the pleural cavity on the diaphragm

**Fig. 3:**
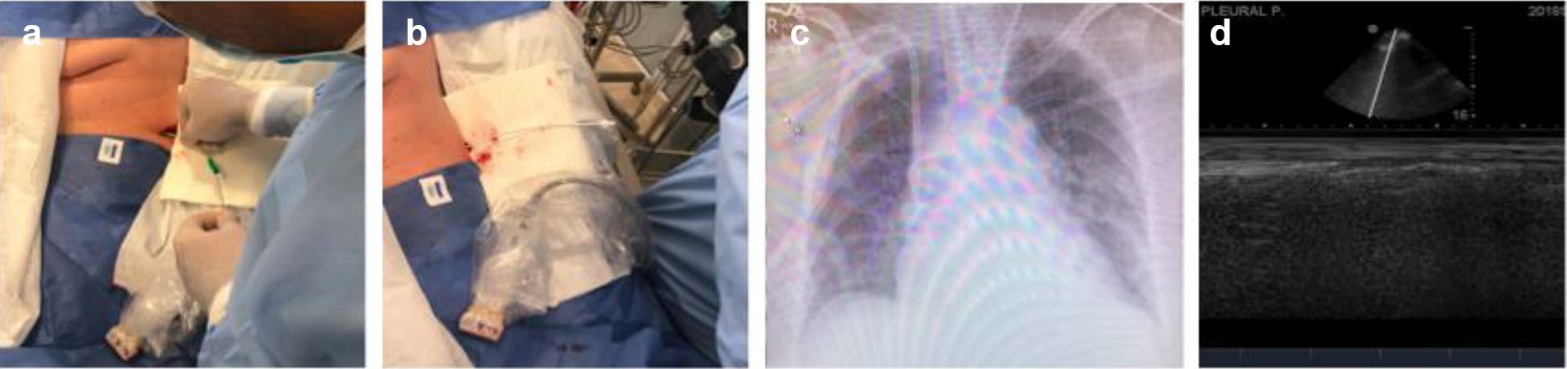
**a** Passage of the dilator (between 1 and 2 cm), depending on thoracic wall thickness. **b** Passage of the pigtail catheter to begin drainage. **c** Chest X-ray after drainage showing a fluid-free pleural cavity. **d** Ultrasound verification, with a sector transducer, of a fluid-free pleural cavity, also dismissing the presence of pneumothorax (“sand-on-the-beach” sign)

The technique used is similar to that described by Vertrugno et al. [22] and was carried out as follows:

For the procedure, a SonoSite M-Turbo ultrasound machine with a 5-MHz small-footprint convex transducer and a 13-MHz linear transducer were used. The real-time ultrasound-guided thoracentesis technique used for the patients included in this study is described below (Figs. 1, 2 and 3):

1. The thoracentesis kit, which contains antiseptic solution, sterile gloves, sterile gauze, local anesthetic, intramuscular needle, subcutaneous needle, 5-, 10-, and 50-ml syringes, 3-way stopcock, 16 gauge thoracentesis needle, 6-French pigtail thoracic drainage catheter, 0.38″ ×80 J-tip metal guide, 6Fr dilator, scalpel, silk to fasten the catheter and collection bag, is prepared.
2. The patient is placed in the supine position with the headboard at 30°.
3. The pleural effusion is visualized with a 5-MHz convex transducer in longitudinal position at the lung base in a posterior axillary line.
4. Once the pleural effusion is visualized, the transducer is rotated to obtain a transverse view.
5. The distance between the visceral pleura and the posterior chest wall is measured.
6. If the distance is greater than 15 mm, the patient is a candidate for the procedure.
7. After asepsis and antisepsis, with the linear transducer in the longitudinal direction, the transducer is positioned on the posterior axillary line in order to obtain an image of the diaphragm between two ribs.
8. Using color Doppler, it is verified that there is no blood vessel interposition.
9. Thoracentesis needle puncture (16 gauge) is performed over the center of the transducer in real time by visualizing needle insertion the entire time.
10. Once the pleural cavity is entered and fluid is collected, the metal guide is inserted, and its position is verified sonographically.
11. Then, the dilator is passed by inserting it 1 to 2 cm, depending on the thickness of the chest wall.
12. The dilator is removed, and the pigtail catheter is inserted over the guide.
13. Once the pigtail catheter is inserted, the drainage system is connected to a 3-way stopcock with one connection directed toward the drainage bag and the other toward a 50-ml syringe. Active drainage is started with the 50-ml syringe, without creating negative pressure that exceeds − 20 cmH_2_O to avoid complications [10]. Importantly, during thoracentesis, the pleural and intrathoracic pressure decrease and the left ventricular afterload increases with a decrease in left ventricular systolic performance, and patients with moderate-to-severe left ventricular dysfunction can develop pulmonary edema [23]. After the procedure, ultrasound is used to determine if residual fluid remains and to rule out the presence of pneumothorax. A chest X-ray performed within 24 h of the procedure is reviewed.

Demographic and clinical variables (macroscopic description of the pleural fluid, amount of drained fluid, interpleural distance, and possible cause), complications and procedure indications were analyzed. The presence of complications was ruled out by performing a lung ultrasound at the end of the procedure and reviewing a chest X-ray performed within 24 h after the thoracentesis. In cases of hemothorax in postoperative cardiac surgery patients, given that there could be confusion as to whether it was residual or associated with thoracentesis, after draining blood from the pleural space, no recurrence of hemothorax after the procedure was verified by ultrasound and on the chest X-ray taken on the following day. The indications for the procedure were classified as diagnostic if the objective of the procedure was only sampling to clarify the etiology of the effusion and as therapeutic if thoracentesis was performed because the patient was considered to have respiratory distress or hypoxemia secondary to the effusion.

A univariate analysis of categorical and continuous variables was performed; normality was tested with the Kolmogorov–Smirnov test. The paired samples *t* test or the Wilcoxon signed rank test was performed. A value of *p* < 0.05 was considered significant. The analysis was performed in STATA 14.

## Results

A total of 81 ultrasound-guided thoracentesis were performed with the described technique. In the study period, 61.7% of the patients were men, and 38.3% were women; the mean age of the observed group was 67 years (SD ± 14 years). In 96% of cases, the indication for thoracentesis was therapeutic, and only 3.7% of patients underwent the procedure as part of their diagnostic algorithm. The diagnoses presented by these patients were, for the most part, postoperative cardiovascular surgery (56.8%), followed by heart failure (16%), pneumonia (12.3%) and others (14.8%), among which paraneoplastic pleural effusion was noteworthy (Table 1).

**Table 1:** Diagnoses presented in ICU patients that led to thoracentesis

	Absolute frequency (*n* = 81)	Relative frequency (*n* = 81)	Accumulated percentage (*n* = 81)
Principal diagnosis			
Cardiovascular surgery	46	56.8	56.8
Heart failure	13	16.0	72.8
Pneumonia	10	12.3	85.2
Other	12	14.8	100.0

The following macroscopic characteristics of the drained fluid were observed: evidence of a hemothorax (55.6%), citrine (29.6%), chylous (6.2%), purulent (7.4%) and paraneoplastic (1.2%). Of the 46 cardiovascular surgery patients who underwent thoracentesis, 38 (82%) had a hemothorax (Table 2).

**Table 2:** Appearance of the pleural fluid drained from the ICU patients

	Absolute frequency (*n* = 81)	Relative frequency (*n* = 81)	Accumulated percentage (*n* = 81)
Appearance of the drainage			
Hemothorax	45	55.6	55.6
Citrine	24	29.6	85.2
Chylous	5	6.2	91.4
Purulent	6	7.4	98.8
Paraneoplastic	1	1.2	100.0

All performed thoracentesis were successful, with one pneumothorax and no hemothorax observed, for a prevalence rate of mechanical complications in this population of 1.2%. The pneumothorax presented in a 77-year-old man not receiving mechanical ventilation, who required diagnostic thoracentesis, had a maximal interpleural distance of 15 mm and, for management, required the passage of a chest tube with drainage of 200 cc of purulent fluid.

**Fig. 4:**
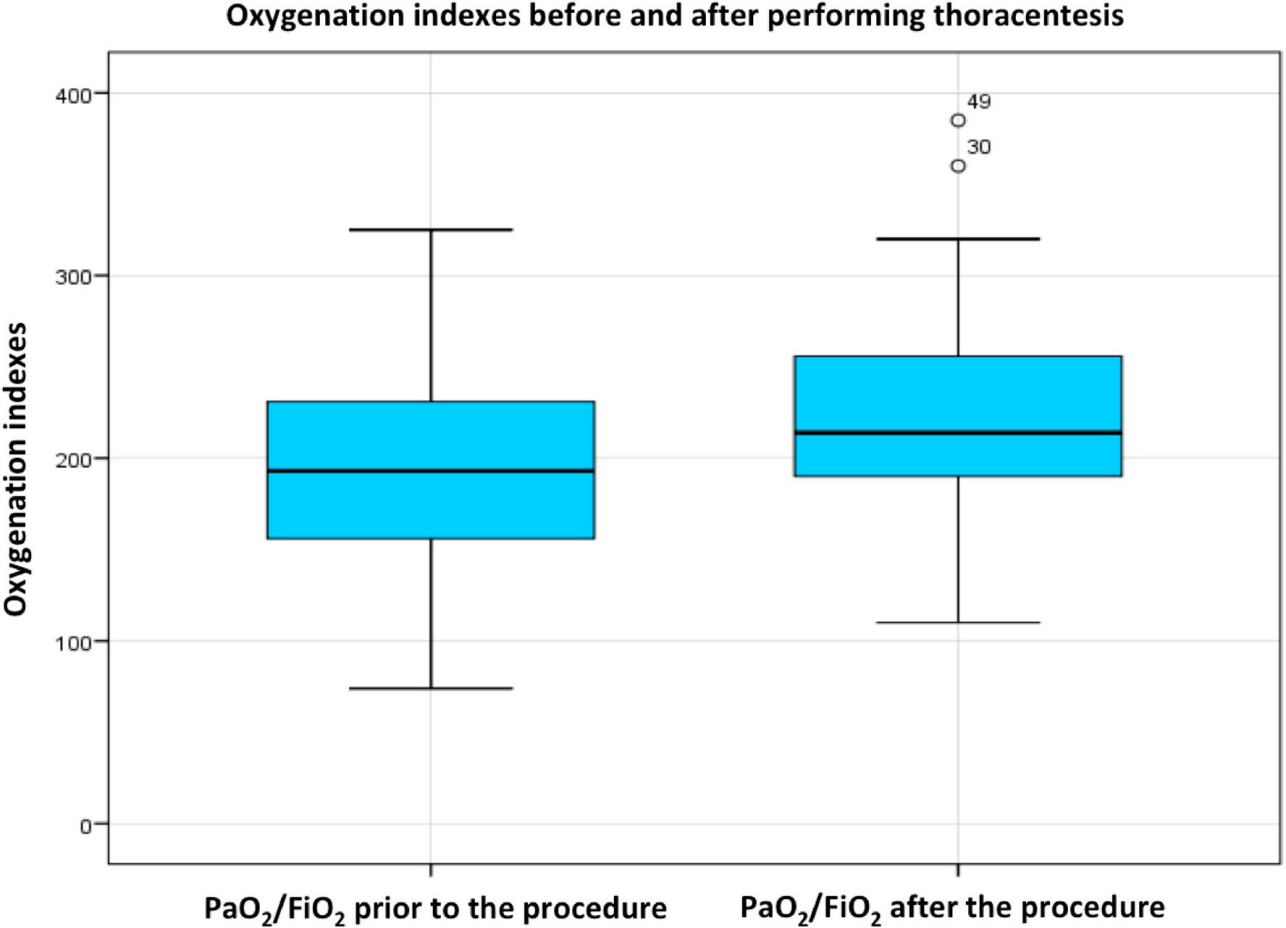
Oxygenation indexes before and after performing thoracentesis. The average PaO_2_/FiO_2_ before the procedure was 198.1, and the average PaO_2_/FiO_2_ after the procedure was 224.6, *p* < 0.05

The mean PaO_2_/FiO_2_ presented positive variations in relation to the measurement at admission (p < 0.05). Prior to the procedure, the PaO_2_/FiO_2_ was 198.1 (SD ± 12.3) (95% CI 184.75–211.45), and after the procedure, it was 224.6 (SD ± 52.9) (95% CI 213.08–226.12) (Table 3, Fig. 4). The stratified analysis showed that this improvement in PaO_2_/FiO_2_ was maintained in the subgroups regardless of the diagnosis (*p* < 0.05).

**Table 3:** Pre- and post-thoracentesis oxygenation parameters in the ICU patients

	Mean (*n* = 81)	Standard deviation (*n* = 81)	Minimum (*n* = 81)	Maximum (*n* = 81)
PaO_2_/FiO_2_ prior to the procedure	198.1	61.3	74.0	385.0
PaO_2_/FiO_2_ after the procedure	224.6	52.9	110.0	385.0
Change in PaO_2_/FiO_2_	29.1	46.4	− 84.0	157.0

Regarding the characteristics of the procedure, drainage was located on the right side in 54.3% and on the left side in 45.7%. Drainage was therapeutic in 96.3% of cases, the median amount drained was 900 ml (95% CI 900–1100 ml), and the pleural effusion volume estimated by the Balik et al. equation was 710 ml (95% CI 600–1000 ml) (Table 4). Spearman’s rho correlation analysis showed a moderate positive relationship between the amount drained and the estimated volume pre-thoracentesis (rho = 0.573) (*p* < 0.05); when differentiating the sides, the correlation between the drained amount and the estimated volume was significant only for the right side (rho = 0.7308, *p* < 0.05), i.e., not for the left side (rho = 0.3776, *p* > 0.05).

**Table 4:** Amount of drainage in milliliters (ml), interpleural distance prior to procedure in millimeters (mm), volume estimated using Balik’s method

	Median (*n* = 81)	Median 95% CI (*n* = 81)	Interquartile range (*n* = 81)
Drained amount (ml)	900	900–1100	550–1100
Distance (mm)	35.5	30–50	28–58.5
Estimated volume (ml)	710.0	600–1000	560–1170
Drained amount, right (ml)	900	650–1000	550–1150
Distance, right (mm)	32.5	26.86–50.83	25–62
Estimated volume, right (ml)	650	537.21–1016.72	500–1240
Drained amount, left (ml)	800	700–947.13	550–1100
Distance, left (mm)	47.5	30–54.95	30–57
Estimated volume, left (ml)	950	600–1099.05	600–1140

Of the total number of patients undergoing thoracentesis, 28.4% were on mechanical ventilation, and the median time to extubation was 6 days (95% CI 5–12 days). For the 23 patients on mechanical ventilation, the PaO_2_/FiO_2_ prior to the procedure was 184.3 (± 48.37) (95% CI 163.39–205.22), with a PaO_2_/FiO_2_ after the procedure of 205.82 (± 57.38) (95% CI 181.01–230.64), maintaining the positive variation but without statistical significance (*p* = 0.08). Of these 23 patients, 11 (47.8%) returned to spontaneous breathing within 7 days after thoracentesis, and in 10 (43.4%), mechanical ventilation was prolonged beyond 1 week. Two patients (8.7%) died in this period. For the 11 patients who achieved early extubation after drainage, the mean PaO_2_/FiO_2_ before and after the procedure was 222.81 (SD ± 40.67) (95% CI 195.49–250.13) and 240.44 (SD ± 50.77) (95% CI 206.33–274.55), respectively. For the 10 patients in whom early extubation was not achieved after drainage, the mean PaO_2_/FiO_2_ values before and after the procedure were 196.40 (SD ± 60.46) (95% CI 153.15–239.65) and 228.12 (SD ± 70.66) (95% CI 177.58–278.60), respectively.

## Discussion

This study shows that real-time ultrasound-guided thoracentesis performed by intensivists at the bedside is a safe procedure, with a prevalence rate of complications of 1.2%, lower than that reported in the literature when blind pleural aspiration is performed, where the presence of pneumothorax ranges from 10 to 18% [12, 24, 25].

The safety of ultrasound-guided thoracentesis has been evaluated in other studies, and it is the recommended technique for this procedure by different international guidelines [12]. Barnes et al. [24] and Gervais et al. [26], in retrospective studies on ultrasound-guided thoracentesis performed by interventional radiologists, reported pneumothorax rates of 4.9% and 2.3%, respectively. Petersen et al. [27] reported a series of 338 ultrasound-guided thoracentesis in the ICU, with four pneumothoraces (1.2%). Lichtenstein et al. [28] and Mayo et al. [29] reported pneumothorax rates in patients receiving mechanical ventilation of 0 and 1.3%, respectively. In these two studies, the procedure was performed in patients under mechanical ventilation. In comparison with those studies, the prevalence of complications reported in the present study is similar or lower.

Another important finding is the impact on oxygenation indexes after pleural drainage, evidencing post-procedure improvement in the PaO_2_/FiO_2_ (*p* < 0.05), in agreement with that reported by Vertrugno et al. [10] and Goligher et al. [30]. In this study, the group of patients on mechanical ventilation was small (28.4%), and of those in whom extubation was achieved in the first 7 days after thoracentesis, the PaO_2_/FiO_2_ post-procedure was higher, but not statistically significant, than in those with prolonged mechanical ventilation. These data suggest that patients in whom drainage led to a greater increase in PaO_2_/FiO_2_ were successfully weaned from mechanical ventilation earlier, as reported by Vertrugno et al. [20]. Further studies are needed to evaluate the importance of oxygenation indices in clinical benefits and outcomes after thoracentesis.

The median volume of drained fluid was 900 ml, and it is known that when volumes greater than 500 ml are drained, oxygenation indices improve [8]. Interestingly, in the postoperative cardiovascular surgery patient subgroup, the benefit of improvement in oxygenation indices was maintained, which is important because it has been reported that the incidence of clinically significant pleural effusion in this group of patients is approximately 7% and in whom almost 50% of the fluid drained is hemorrhagic [4].

Spearman’s rho correlation analysis showed a moderate positive linear relationship (rho = 0.573) between the drained amount and the estimated volume; however, this correlation was lower than that described by Balik et al. [16], which can be explained by the fact that in this study, the estimate was applied to patients with spontaneous breathing and with the headboard at 30°. However, when analyzing each side separately, the correlation between the drained amount and the estimated volume was significant only on the right side (rho = 0.7308) and not on the left side (rho = 0.3776), which can be explained by the presence of the heart, as it increases the pleural effusion level, leading to an overestimation of the calculated volume [17, 18].

The intensivists who performed thoracentesis during this study have more than 5 years of experience in the use of ultrasound at the bedside, and during their residency program, they were trained to perform ultrasound-guided procedures, which helps to explain the low rate of complications reported in this study. To begin performing thoracentesis in real scenarios, we first recommend the development of this skill in phantom models of lung anatomy [31].

This study has the strength of being the first series in which the majority of thoracentesis procedures were performed under ultrasound guidance in real time by intensivists, confirming the safety of this technique and the improvement in oxygenation indices.

This study is observational and has limitations. A lung ultrasound was not performed on all ICU patients during the study period; therefore, the proportion of pleural effusions that required drainage could not be reported. This study also does not allow assessing the safety of thoracentesis performed by other ultrasound-guided techniques, such as radiological marking and subsequent drainage by a different physician or marking and immediate puncture.

## Conclusions

Real-time ultrasound-guided thoracentesis performed by intensivists is a safe procedure with a low complication rate compared to that for blind techniques, has a complication rate similar to that for other ultrasound-guided techniques and leads to significant improvement in oxygenation rates. Future studies are needed to determine the impact of these results on outcomes such as mortality, length of ICU stay and days of mechanical ventilation.

## Data Availability

All data used and analyzed during this study are available to be sent by email at the request of the editorial committee, anonymously and coded.

## Abbreviations

ICU: Intensive care unit
SD: Standard deviation
ml: Milliliters
mm: Millimeters
PaO_2_/FiO_2_: Partial pressure arterial oxygen/fraction of inspired oxygen
Sep: Maximal interpleural distance
Vpl: Pleural fluid volume
